# Supplementary figures and images for: The NF-κB regulator Bcl-3 restricts terminal differentiation and promotes memory cell formation of CD8+ T cells during viral infection
Source: PLoS Pathog. 2021 Jan 28;17(1):e1009249. doi: 10.1371/journal.ppat.1009249 (PMC7872245; doi:10.1371/journal.ppat.1009249)

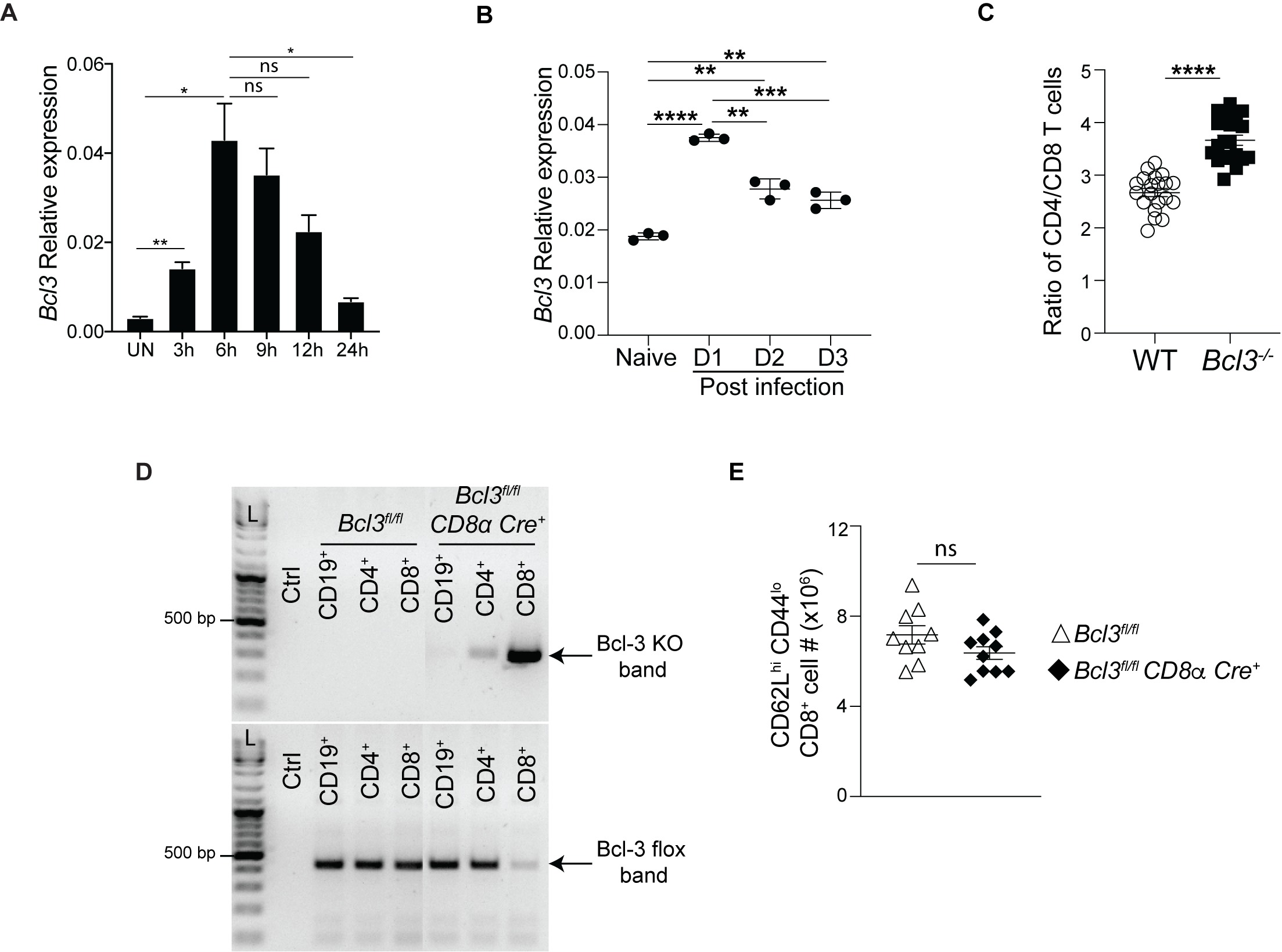

Supplement: S1 Fig — (A) Bcl3 mRNA levels in purified CD8+ T cells upon CD3 and CD28 stimulation. n = 4 mice. (B) Bcl3 mRNA levels in bead-purified CD8+ T cells from spleens of uninfected and LCMV Armstrong-infected WT mice (C57BL/6). n = 3 mice for each time point. (C) Ratio of CD4/CD8 T cells in blood of uninfected mixed bone marrow chimeric mice after at least 6 weeks of reconstitution. Data is representative of three independent experiments. n = 20 mice. (D) Knock down efficiency of Bcl3 in CD8α Cre mice. Bcl3-Flox and knock-out bands were checked by qPCR from DNA of sorted cells of specific genotypes, as shown. (E) Spleens of uninfected Bcl3fl/fl and Bcl3fl/fl CD8α Cre+ mice were analyzed for CD8+ T cell subsets. n = 9–10 mice for each genotype. Error bars represents SEM. *p<0.05, **p<0.01, ***p<0.001, ****p<0.0001. ns = not significant. (TIF) [file ppat.1009249.s001.tif]

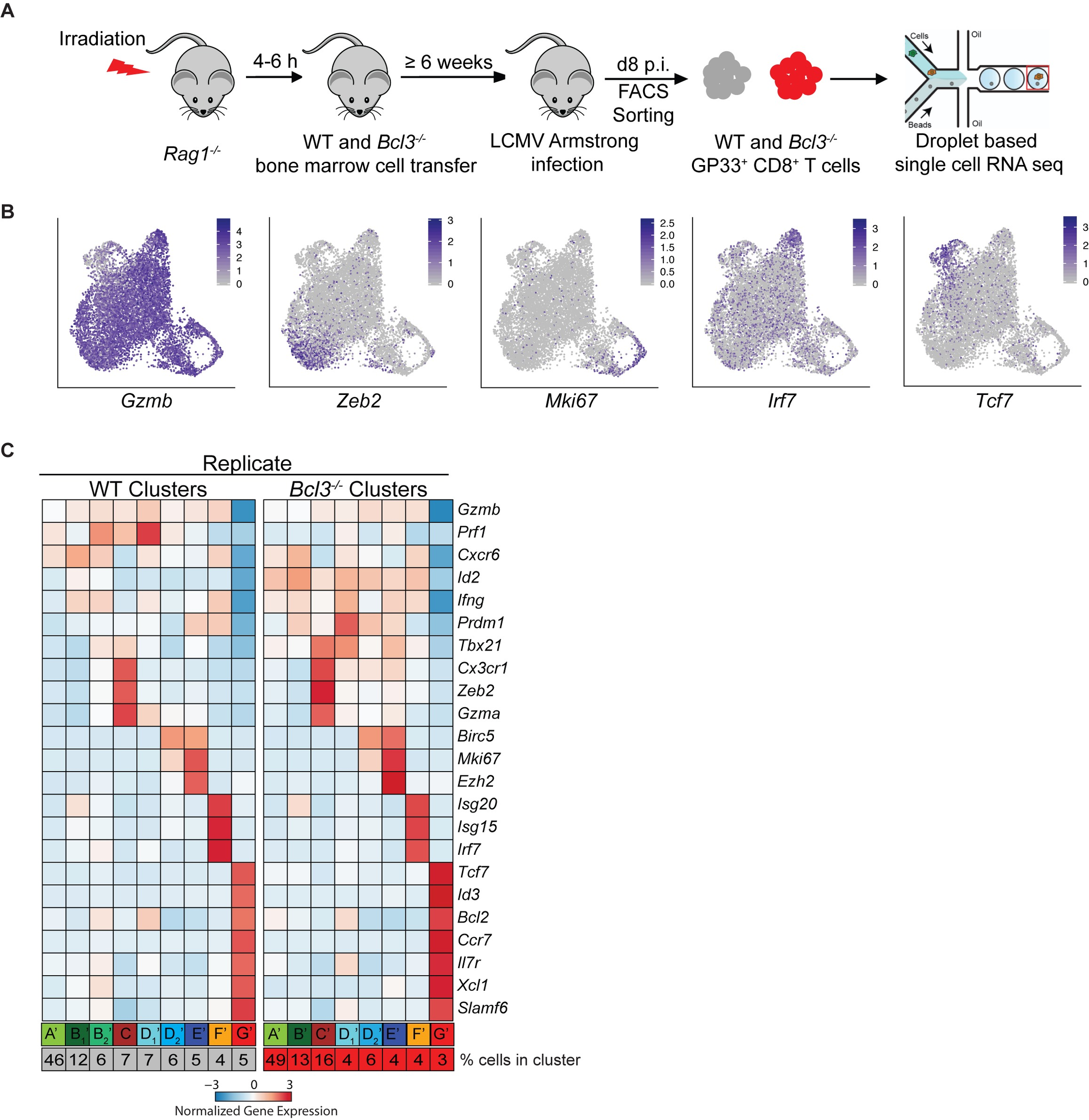

Supplement: S2 Fig — (A) Experimental protocol: At least 6 weeks post reconstitution, mixed bone marrow chimeric mice were infected with LCMV Armstrong. At 8 d p.i., GP33 tetramer+ CD8+ WT or Bcl-3 KO cells were sorted and subjected to single cell RNA sequencing. (B) UMAP plots show the relative expression of the indicated genes in Bcl-3 KO and WT cells from the experiment shown in Fig 2. (C) Heatmap shows row-standardized expression of selected genes among clusters of Bcl-3 KO and WT cells in an independent experiment performed with another mixed bone marrow chimera and processed as described in Fig 2. For each genotype, percentages indicate the size of individual clusters relative to the total population. Cluster A’, B1’/ B2’, C’, D1’/D2’, E’, F’ and G’ of the replicate set are roughly equivalent to clusters A, B, C, D, E, F and G of the first set in Fig 2, respectively (in this analysis the program distinguished more effector subsets). (TIF) [file ppat.1009249.s002.tif]

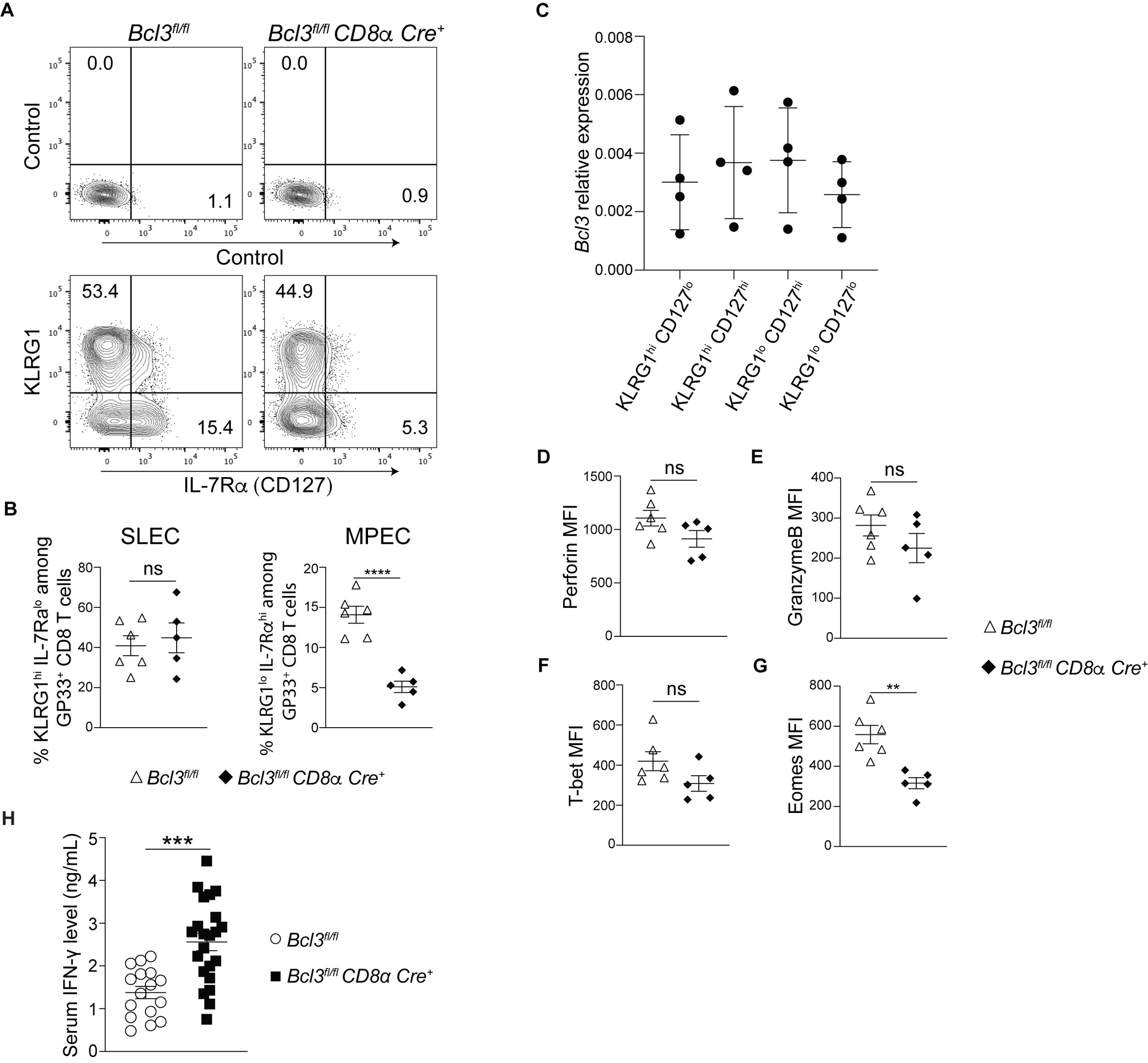

Supplement: S3 Fig — (A and B) GP33-epitope specific CD8+ T cells from spleens of Bcl3fl/fl and Bcl3fl/fl CD8α Cre mice were analyzed for SLEC and MPEC subsets on day 8 post-acute LCMV infection. n = 5–6 mice for each genotype. (C) Bcl3 mRNA levels in various effector subsets of WT CD8+ T cells specific for any of three LCMV epitopes (GP33, GP276, NP396), at day 8 post LCMV Armstrong infection. n = 4 mice. (D-G) GP33-epitope specific spleen CD8+ T cells from Bcl3fl/fl and Bcl3fl/fl CD8α Cre mice were analyzed for the indicated effector markers by flow cytometry at day 8 post-acute LCMV infection. n = 5–6 mice for each genotype. (H) Serum IFN-γ levels on day 5 post-acute LCMV infection. Data are cumulative from 3 independent experiments. Error bars represent SEM with n = 16–22 mice for each genotype. **p<0.01, ***p<0.001, ****p<0.0001, ns = non-significant. (TIF) [file ppat.1009249.s003.tif]

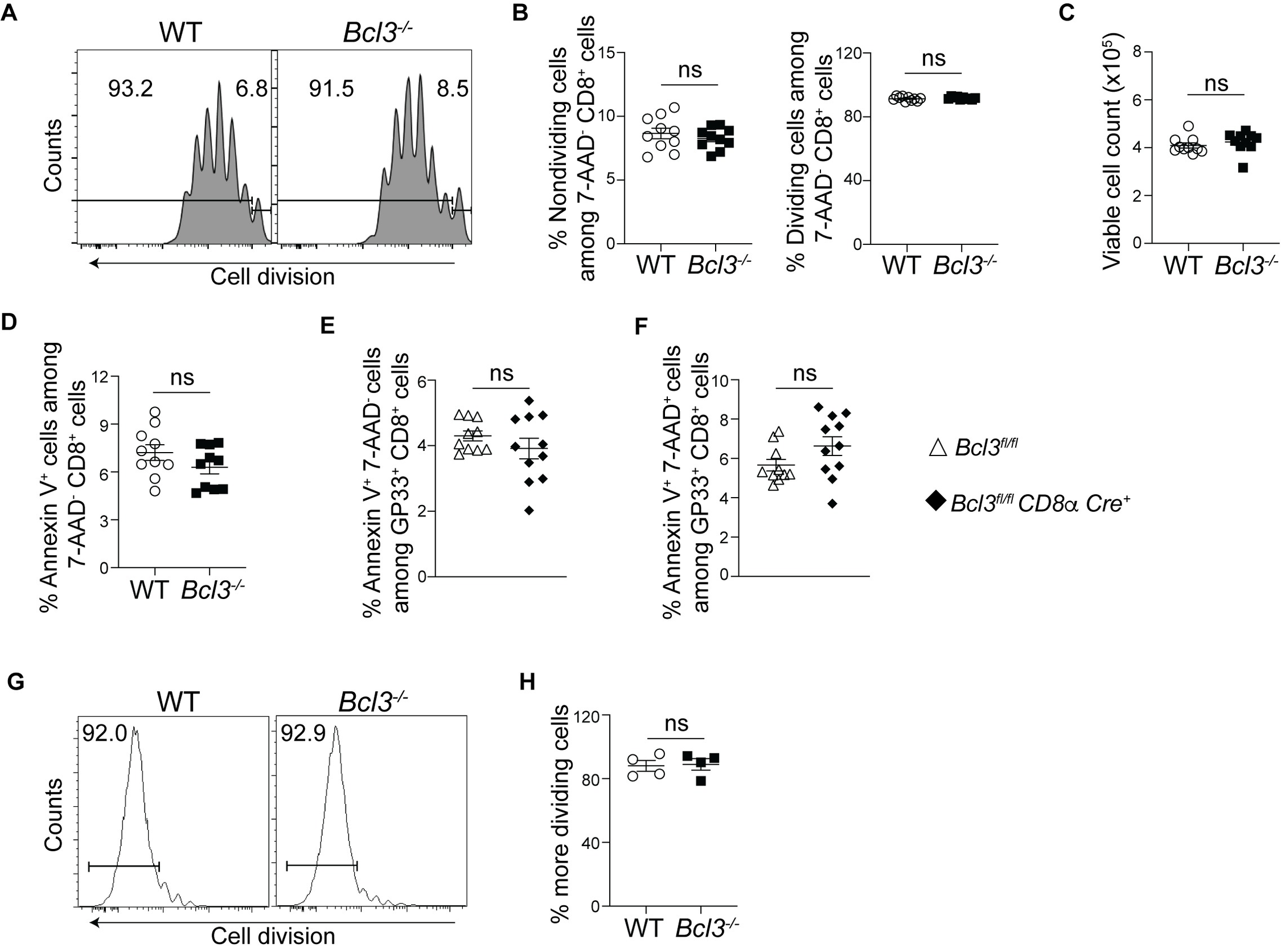

Supplement: S4 Fig — (A-D) 2 x 10^5^ cell trace violet-stained purified CD8+ T cells from Bcl3-/- and control mouse spleen were cultured for 72 hours in the presence of plate-bound anti-CD3ε (2 μg/mL) plus soluble anti-αCD28 (1 μg/mL) along with murine IL-2 (10 ng/mL). Data are averages of two independent experiments with n = 5 for each genotype. (A) Representative plot for cell division. (B) Percentages of nondividing and dividing cells. (C) Number of live cells by trypan blue staining. (D) Percentages of annexin V+ (apoptotic) cells. (E and F) GP33-epitope specific CD8+ T cells from spleens of Bcl3fl/fl and Bcl3fl/fl CD8a Cre mice were analyzed on day8 post-acute LCMV infection. Data are averages of two independent experiments with n = 10–11 mice for each genotype. (E) Percentages of annexin V+ 7AAD- cells (apoptotic cells) among H-2Db-GP33+ CD8+ T cells. (F) Percentages of annexin V+ 7AAD+ cells (dead cells) among H-2Db-GP33+ CD8+ T cells. (G and H) 1x10^6^ cell trace violet-stained purified WT or Bcl3-/- CD8+ T cells from P14 transgenic mice were adoptively transferred to congenic recipient mice. On the next day, mice were infected with LCMV Armstrong and mesenteric lymph nodes were analyzed 4 days after infection. n = 4 mice in each group. (G) Representative plot for cell division. (H) Percentages of more dividing cells as gated in G. Error bars indicate SEM. ns = non-significant. (TIF) [file ppat.1009249.s004.tif]

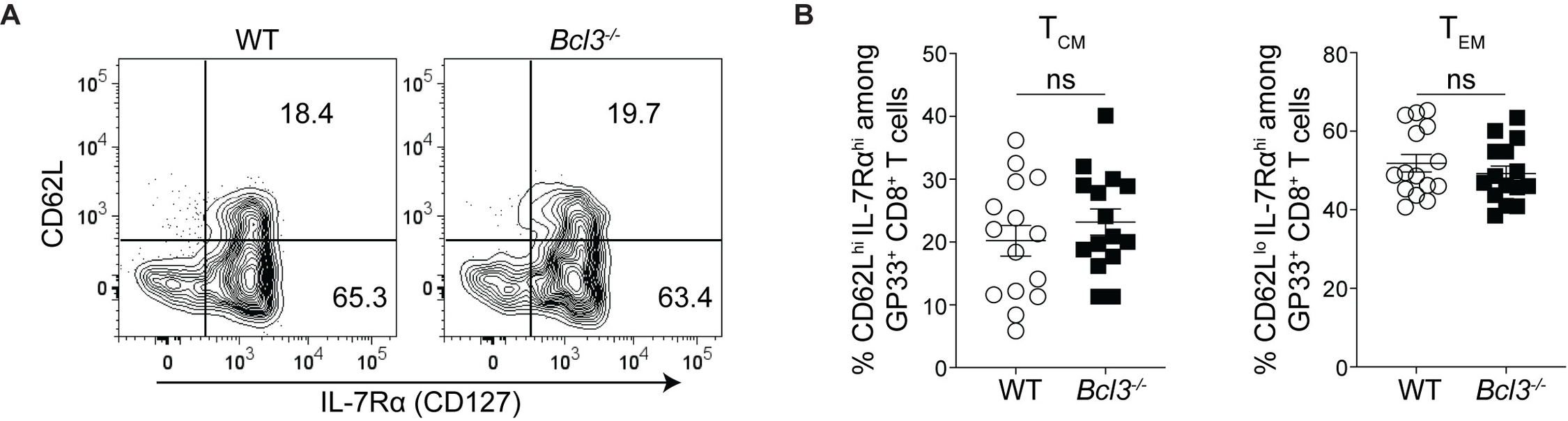

Supplement: S5 Fig — (A and B) 8 weeks post LCMV Armstrong infection mixed bone marrow chimeras were analyzed for TCM and TEM subsets in spleen. (A) Representative contour plots show TCM (CD62Lhi IL-7Rαhi) and TEM (CD62Llo IL-7Rαhi) subsets among H-2Db-GP33+ CD8+ T cells. (B) Graph summarizes data from three independent experiments with error bars representing SEM. n = 15 mice for each CD8+ T cell genotype. ns = not significant. (TIF) [file ppat.1009249.s005.tif]

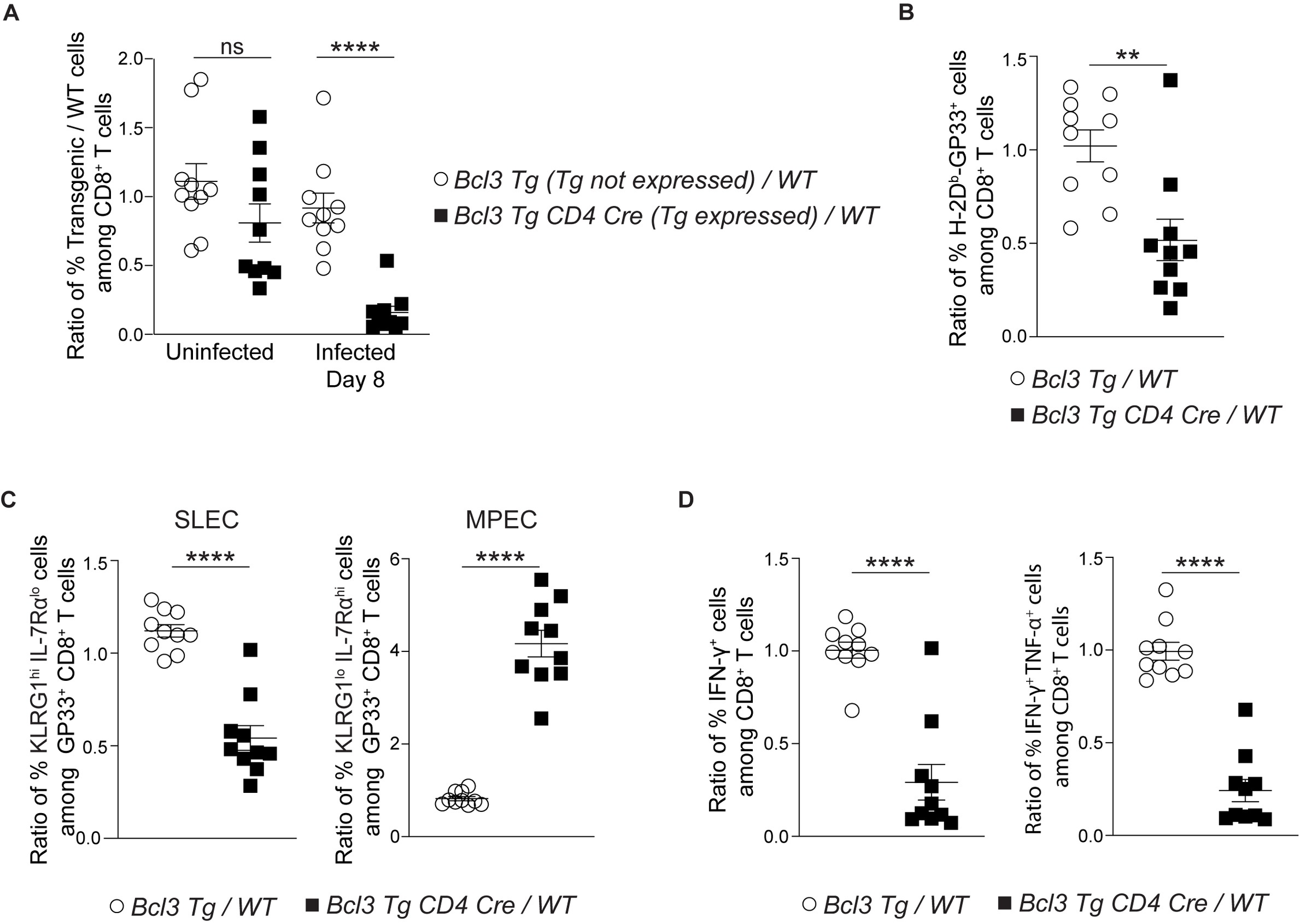

Supplement: S6 Fig — (A) After at least 6 weeks of reconstitution, ratio of proportions of Bcl3 transgene non-expressing or expressing cells to WT cells among CD8+ T cells in mixed bone marrow chimeric mice before infection (in blood) or at day 8 of LCMV Armstrong infection (spleen). Graph summarize data from two independent experiments with n = 10 mice each group. (B-D) After at least 6 weeks of reconstitution, mixed bone marrow chimeras were infected with LCMV Armstrong and spleens were analyzed 8 d p.i. Graphs summarize data from two independent experiments with n = 10 mice each group. (B) Ratio of proportions of GP33 tetramer+ cells among CD8+ T cells from chimeric mice containing a non-expressing Bcl-3 transgene or containing a Bcl-3 expressing transgene. (C) Ratio of proportions of KLRG1hi IL-7Rαlo cells (SLEC, left graph) and KLRG1lo IL-7Rαhi cells (MPEC, right graph) among GP33+ CD8+ T cells from chimeric mice containing a non-expressing Bcl-3 transgene or containing a Bcl-3 expressing transgene. (D) Ratio of proportions of IFN-γ+ cells (left graph) and IFN-γ+ TNF-α+ cells (right graph) among CD8+ T cells from chimeric mice containing a non-expressing Bcl-3 transgene or containing a Bcl-3 expressing transgene upon ex vivo stimulation of splenocytes with GP33 peptide. Error bars represents SEM. **p<0.01, ****p<0.0001. ns = not significant. (TIF) [file ppat.1009249.s006.tif]
