# Supplementary material for: The NF-κB regulator Bcl-3 restricts terminal differentiation and promotes memory cell formation of CD8+ T cells during viral infection
Source: PLoS Pathog. 2021 Jan 28;17(1):e1009249. doi: 10.1371/journal.ppat.1009249 (PMC7872245; doi:10.1371/journal.ppat.1009249)
Supplement: Attachment — Submitted filename: Point by point response.docx [file ppat.1009249.s007.docx]

Point by point reply to Editor and Referees’ comments, “The NF-κB regulator Bcl-3 restricts terminal differentiation and promotes memory cell formation of CD8^+^ T cell in viral infection” (PPATHOGENS-D-20-01573).

We thank the editors and referees for their interest in our study and for their constructive criticisms. We have addressed the editor and referees’ comments by adding new analyses, shown in Figs. S1B, S1E, S3 and S4 and introducing changes in the results section (red colored text in the revised manuscript).

**Editors’ comments:**

a) Show whether Bcl3 is expressed on different CD8 T cell subsets during LCMV infection.

We have addressed this point with new experiment shown in Fig. S1B and presented lines 150-151 of the manuscript. Because there are no Bcl-3 antibodies suitable for intra-cellular staining and flow cytometry, we answered this question by assessing *Bcl3* RNA expression by qRT-PCR. *Bcl3* was rapidly and transiently upregulated in splenic CD8^+^ T cells with a peak by Day 1 post infection. We performed the subset analysis at day 8 post infection, when SLEC and MPEC subsets can be distinguished and detected Bcl-3 expression in all subsets. These new data have been added as Fig. S3C and is discussed in the text lines 220-222 of the manuscript.

b) Demonstrate if there is differential proliferation and or apoptosis of WT and Bcl3^-/-^ CD8 T cells.

We have addressed this point with new experiments shown in Fig. S4 and presented lines 237-245 of the manuscript, including *in vivo* adoptive transfer of CD8^+^ T cells from LCMV-specific TCR transgenic (P14) mice, and *in vitro* TCR stimulation experiments. No proliferation or apoptosis differences were observed between WT and *Bcl3* KO CD8^+^ T cells in either system.

c) Show the expression of perforin, gran B, Tbet, Eomes and other effector molecules of CD8 T cells in WT and Bcl3^-/-^ mice.

We have addressed this point with new experiments shown in Fig. S3D-S3G and is discussed in the text lines 229-233 of the manuscript. A significant difference between WT and KO cells from infected mice was only observed for Eomes.

d) Provide control gates for Fig2 C/D FACS plots

We have addressed this point with new experiment using Bcl-3 conditional knock out mice. New data has been shown in Fig. S3A and S3B and is discussed in the text line 219 of the manuscript. New figure shows the control gates for KLRG1 and IL-7Rα.

**Reviewer #1:**

In this manuscript, authors have characterized the role of Bcl-3 in terminal differentiation of memory CD8+ T cells. Using acute LCMV model, investigators show that BCL3 is critical for maintaining balance of terminal effector/memory cell balance during CD8+ T cell differentiation. This study lacks mechanistic details for observed differences in SLECs and MPECs post LCMV infection and recall responses. Thus, descriptive phenotypic changes in Knock-out mice in the absence of mechanistic details diminishes the importance of these findings. Nevertheless, overall findings are interesting and various conclusions are well supported by data generated using WT/Bcl-3 KO bone-marrow chimera and CD8-specific conditional Bcl-3 knock-out mice.

We are gratified by the referee's interest in our findings and positive comments on the approaches used in our study. We provide new data (as discussed below in specific comments) that address concerns expressed by the referee.

**Specific comments**

1. Authors show transient expression of Bcl-3 in CD8+ T cells post CD3/CD28 stimulation. Although this data indicates the transient induction of Bcl-3 post TCR stimulation, similar expression kinetics data for Bcl-3 in naïve, effector/memory CD8+ T cells post LCMV infection is critical as a premise to study the role of Bcl-3 in in vivo differentiation of CD8+ T cells post LCMV infection.

We have addressed this point with new experiment shown in Fig. S1B and presented lines 150-151 of the manuscript. Because there are no Bcl-3 antibodies suitable for intra-cellular staining and flow cytometry, we answered this question by assessing *Bcl3* RNA expression by qRT-PCR. *Bcl3* was rapidly and transiently upregulated in splenic CD8^+^ T cells with a peak by Day 1 post infection. We performed the subset analysis at day 8 post infection, when SLEC and MPEC subsets can be distinguished and detected Bcl-3 expression in all subsets. These new data have been added as Fig. S3C and is discussed in the text lines 220-222 of the manuscript.

2. Mixed bone-marrow chimera experiments do not show overall differences in LCMV-specific CD8+ T cell responses after acute infection. Further, mixed chimera experiments do not rule out the role for Bcl-3 in other myeloid cells particularly APCs in regulation of effector versus memory T cell differentiation. Although authors have used conditional knock-out mice for CD8+ T cells to demonstrate cell-intrinsic role of Bcl-3 in efficient formation of memory CD8+ T cells, the choice of cre-strain indicate that there could be developmental defects in CD8+ T cells this conditional knock-out mice.

We agree that each experimental approach has limitations that must be considered in drawing conclusions. However, the correspondence of the results using both CD8-specifc knockout of Bcl-3 and mixed chimeras provides consistent evidence in support of the hypothesis. Regarding CD8^+^ T cell development, we provide new data (Fig S1E, lines 173-175) showing similar number of naïve CD8^+^ T cells in spleen of *CD8α Cre* conditional KO and control mice. Indeed, as mentioned in the results section (line 171-173), expression of Cre in these mice is driven by a specific CD8 enhancer E8(I) (reference 35) and is limited to peripheral mature CD8^+^ T cells. Both the original study identifying this enhancer (reference 36) and another study (by one of us, RB, *J Exp. Med. 2007; 204(2):267-72*) that generated an independent transgenic line driven by this enhancer, have found that E8(I) expression is limited to mature, post-selection CD8 SP thymocytes and post-thymic CD8^+^ T cells. We suggest that the new Fig. S1E and the previous work cited above are sufficient to exclude an effect of CD8α-Cre deletion of Bcl3 during T cell development. Furthermore, non-lymphoid cell expression of the E8(I) enhancer has not been reported. Upon LCMV infection, naïve cells are believed be the major driver of anti-viral responses. In figure Fig 1E, we have shown that at day 8 post-infection virus epitope GP33-specific CD8^+^ T cells were comparable between control (*Bcl3^fl/fl^*) and conditional knock-out mice (*Bcl3^fl/fl^ CD8α Cre*). Hence, the results on Bcl-3-dependent memory cell formation are unlikely to reflect a confounding developmental defect.

3. There is no clear mechanistic explanation for increased terminal effector cells over MPECs in Bcl-3-/- mice studies. Is this because of increased proliferation or increased survival/reduced apoptosis? Also, mixed bone marrow-chimera experiment show differential reconstitution of CD4/CD8 T cells in WT and KO cells. This data points initial differences in WT and KO cells could drive differential CD8+ T cell responses post LCMV challenge.

We have addressed this point with new experiments shown in Fig. S4 and presented lines 237-245 of the manuscript, including *in vivo* adoptive transfer of CD8^+^ T cells from LCMV-specific TCR transgenic (P14) mice, and *in vitro* TCR stimulation experiments. No proliferation or apoptosis differences were observed between WT and *Bcl3* KO CD8^+^ T cells in either system. To rule out effects caused by differential reconstitution of CD4/CD8 T cells in the mixed bone marrow chimera model, we also used *CD8α Cre* conditional knock mice where Cre expression is highly limited to peripheral CD8^+^ T cells (ref 35-37). Experiments with both mouse models leads to similar conclusions. Hence, observations made are indeed because of loss of Bcl-3 in CD8^+^ T cells.

4. Data represents multiple experiments. Did authors pool data from different experiments? If yes, was there a similar statistical difference for each individual experiment?

We pooled data from multiple experiments and the number of experiments for each figure where data were pooled is given in the corresponding legends. Although differences between Bcl3-sufficient and -deficient mice were consistent across individual experiments comprising each pool, statistical analysis was performed only after pooling the data.

**Reviewer #2**

This study is very interesting as it shows the role of Bcl3 in generation of cd8 memory t cells. The study is well designed and uses various high end techniques and tools to make a conclusion.

We thank the referee for these positive comments and for her or his interest in our study.

1. Although this study identify the role of Bcl3 in generation of CD8 memory response, it is not clear as to why bcl3 regulate the generation of MPECs. Whether bcl3 somehow controlling activation induced cell death and therefore as a result a reduced frequency of MPECs were found in bcl3 deficient mice. Authors must address this.

We have addressed this point with new experiments both *in vitro* and *in vivo* as shown in Fig. S4 and presented lines 237-245 of the manuscript. No apoptosis differences were observed between WT and *Bcl3* KO CD8^+^ T cells in either system.

2. In addition, authors should show whether Bcl3-/- cd8 t cells proliferate less both in vitro and in vivo. Authors should also show the expression of perforin, gran B, Tbet, Eomes and other effector molecules of CD8 T cells in wt and Bcl3-/- mice.

We have addressed this point with new experiments both *in vitro* and *in vivo* as shown in Fig. S4 and presented lines 237-245 of the manuscript. No proliferation differences were observed between WT and *Bcl3* KO CD8^+^ T cells in either system.

We performed new experiment to assess expression of perforin, granzyme B, T-bet and Eomes as shown in Fig. S3D-S3G and is discussed in the text lines 229-233 of the manuscript. A significant difference between WT and KO cells from infected mice was only observed for Eomes.
